# Public health actions in response to pathogen detection in wastewater and the environment: a scoping review

**DOI:** 10.3389/fpubh.2025.1675742

**Published:** 2026-01-16

**Authors:** Maarten de Jong, Jolinda de Korne-Elenbaas, Ewout Fanoy, Gertjan Medema, Miranda de Graaf, Maria Prins, Maarten F. Schim van der Loeff, Joost Daams, Ana Maria de Roda Husman, Janneke C. M. Heijne

**Affiliations:** 1 Department of Infectious Diseases, Public Health Service Amsterdam (GGD Amsterdam), Amsterdam, Netherlands; 2 Internal Medicine, Amsterdam Institute for Immunology and Infectious Diseases (AI&I) and Amsterdam Public Health Research Institute (APH), Amsterdam UMC, University of Amsterdam, Amsterdam, Netherlands; 3 Swiss Federal Institute of Aquatic Science and Technology, Eawag, Dübendorf, Switzerland; 4 Department of Infectious Diseases, Public Health Service Region Utrecht (GGD Regio Utrecht), Utrecht, Netherlands; 5 KWR Water Research Institute, Nieuwegein, Netherlands; 6 Department of Viroscience, Erasmus University Medical Center, Rotterdam, Netherlands; 7 Medical Library, Amsterdam UMC Location University of Amsterdam, Amsterdam, Netherlands; 8 Centre for Infectious Disease Control (CIb), National Institute for Public Health and the Environment (RIVM), Bilthoven, Netherlands; 9 Institute for Risk Assessment Sciences, Utrecht University, Utrecht, Netherlands

**Keywords:** infectious agents detection, public health response, wastewater analysis methods, wastewater sampling methods, wastewater surveillance, wastewater monitoring

## Abstract

**Introduction:**

Rapid detection of infectious disease agents is crucial for timely public health responses. Wastewater and environmental surveillance (WES) offers a complementary approach by detecting pathogens shed by infected individuals, including asymptomatic cases. This scoping review provides an overview of reported public health actions in response to WES for human pathogens. It also summarizes sampling and analysis methods and offers insights for future implementation.

**Methods:**

The protocol for this review was registered in the PROCEED open-access registry. A systematic search was conducted in MEDLINE, EMBASE, and Web of Science for peer-reviewed literature published up to 31 July 2024. Studies were included if they reported public health actions in response to WES related to infectious diseases in human populations. Two reviewers independently screened studies and extracted data on public health responses, sampling, and analytical methods.

**Results:**

Of the 6,630 articles screened, 49 met the inclusion criteria. Most studies (92%) were published between 2021 and 2024, with SARS-CoV-2 as the primary focus (82%), followed by poliovirus (16%). Research was largely conducted in high-income regions: North America (51%), Asia (22%), and Europe (14%). Target populations included urban residents (57%) and on-campus students (31%) and local authorities were more often involved in WES efforts than national agencies (51% vs. 33%). In 75% of studies, at least two public health actions were implemented, and 20% reported five or more. The most common actions related to reactive disease control (*n* = 69), including testing, isolation, and contact tracing. Proactive disease control actions (*n* = 33) and public health communication (*n* = 22) were also described. Weekly sampling (57%) and composite methods (67%) were most used. Manhole sampling, despite equal frequency with treatment plant sampling (35%), led to significantly more public health actions (61 vs. 35). Long-term surveillance was often reported but rarely sustained. Quantitative and molecular analyses dominated; sequencing was rarely used (4%).

**Conclusion:**

While reporting on public health actions following WES remains limited, this review illustrates its potential to inform timely, local interventions. Future studies should broaden pathogen targets, embed public health action planning in study design, and expand WES use in low-resource settings.

## 1 Introduction

Effective and rapid detection of infectious disease agents during an outbreak is important to enable targeted public health interventions. Timely data generation enables actionable opportunities to limit the spread, severity, and duration of potential outbreaks. To enable such a response, surveillance and monitoring systems are essential (1, 2). Traditional surveillance methods usually rely on clinical data, such as case reports, hospitalizations or diagnostic testing. These methods are effective but can be hindered by patients not having access or facing barriers to healthcare, delays in data collection and reporting, and the common absence of diagnostics for mild disease (3–5). Additionally, individuals might not seek medical attention for mild symptoms, further limiting detection and reporting (6). Therefore, innovative approaches are sought to enhance our ability to detect and respond to potential outbreaks in both high- and low-resource settings (7–10). Wastewater and environmental surveillance (WES) is such an approach (11, 12). It enables monitoring of pathogens by collecting samples from wastewater systems, offering a non-invasive way to observe presence and varieties of pathogens within populations (13). WES complements traditional approaches and enables early detection of pathogens, even in asymptomatic cases or before symptoms or clinical cases are reported (14–17).

It has been shown that WES is a valuable tool for detecting and monitoring pathogens like poliovirus (18–20), norovirus (21, 22), hepatitis E virus (23), and other infectious agents in communities (24). It has led to public health actions such as targeted vaccination campaigns in specific areas or populations and to assessment of their impact (25–27). The COVID-19 pandemic has significantly advanced WES worldwide, demonstrating its value as a cost-effective, rapid, and reliable method for tracking the spread of severe acute respiratory syndrome coronavirus 2 (SARS-CoV-2) and its variants within populations (28–30). This has led to various recent reviews about the practical and technical use of WES for outbreak management and control (31–33). To our knowledge, a comprehensive and detailed overview of public health responses to pathogen detection in wastewater is currently lacking.

This scoping review aims to assess the potential of WES in infectious disease control by providing an overview of public health actions taken in response to pathogen detection in wastewater and their corresponding sampling strategies and analysis methods. Moreover, based on the findings further improvements in the use of WES are suggested.

## 2 Methods

The protocol for this scoping review was registered in the PROCEED open access registry (PROCEED-24-00222) (34), and published in medRxiv (35). We report this scoping review in accordance with the Preferred Reporting Items for Systematic reviews and Meta-Analyses extension for Scoping Reviews (PRISMA-ScR)-checklist (see Supplementary Data Sheet 1) (36).

### 2.1 Definitions

We defined “pathogen detection in wastewater” as the process of identifying genomic material of pathogens (bacteria, viruses, fungi and parasites) in wastewater. Informed by the World Health Organization's (WHO) Essential Public Health Functions (37), we defined “public health actions” as the efforts aimed at infectious disease management and control to improve population-level health outcomes, while reducing risks and promoting health at the individual level. We defined “wastewater” as untreated wastewater containing a mixture of human waste and other (domestic) waste. “Wastewater sampling” was defined as the process of collecting wastewater samples from one or more locations for analysis. We defined “wastewater analysis” as the examination and assessment of the composition and characteristics of wastewater to identify genetic markers of pathogens.

### 2.2 Information sources and search strategy

A comprehensive search was performed in the databases MEDLINE, Embase, and Web of Science from 01 January 2014 to 31 July 2024 in collaboration with a medical information specialist (JD). All literature published before 1 January 2014 was excluded since WES has advanced significantly over the past decade through technical innovations, standardized methods and its integration into public health responses, particularly during the COVID-19 pandemic. The search was conducted on title, abstract and keywords and search language was restricted to English. The search string consisted of three key concepts: wastewater, pathogens and public health. See Supplementary Data Sheet 2 for the detailed search string.

### 2.3 Eligibility criteria

We included literature in the field of human infectious diseases dealing with wastewater sampling and analysis, with an explicit link to public health actions. When needed, we consulted the WHO's Essential Public Health Functions to evaluate whether the described public health actions aligned with the criteria and to determine whether the record should be included in the scoping review. All literature not written in English and not peer-reviewed was excluded. Systematic reviews, commentaries, editorials and letters to the editor were also excluded as they do not provide peer-reviewed primary data.

### 2.4 Selection of sources of evidence

After deduplication, all search results were imported to and managed with Rayyan software, a web-based systematic review tool that assists in expediting the screening phase (38). Two authors (MJ and JK) performed screening of titles and abstracts independently by applying the eligibility criteria. After pilot-testing 300 articles, they discussed the similarities and differences in their inclusion and exclusion decisions, after which they proceeded to screen the remaining articles. Subsequently, they independently conducted full-text reviews of included literature to determine eligibility. In both steps, disagreements were resolved by discussions between the two authors and if needed, by a third senior reviewer (JH). The results of the search and the study inclusion process are presented in a PRISMA flow diagram (39).

### 2.5 Data extraction

Two reviewers (MJ and JK) extracted data independently from the eligible full-text articles in an Excel sheet. Discrepancies were discussed until consensus was reached. Unresolved discrepancies were arbitrated by senior reviewers (JH and AMRH). Authors of articles were consulted for additional information and clarification where necessary during the extraction process. The data extraction form was initially developed based on input from all members of the research team, each contributing insights from their respective areas of expertise. Categorization of the extracted data was also developed and refined in this manner. WES catchments often encompass a broad spectrum of individuals, from permanent residents to transient populations. In this review we extracted detailed information from the included studies regarding the specific populations targeted within each WES catchment. This allowed us to provide more precise descriptions of the populations covered. When needed, the WHO's Essential Public Health Functions framework was used as guidance to formulate public health actions that aligned with the specific infectious disease control and management scope of this review. It was ensured that overlap between the categories of public health actions was minimized so that each category can be regarded as a clearly delineated public health action. The data extraction form was subsequently refined during the review process and resulted in the final data extraction table (Supplementary Table 1).

### 2.6 Data synthesis

A descriptive synthesis approach was used to summarize the extracted data. Study characteristics and reported outcomes were organized and analyzed using Microsoft Excel for descriptive and exploratory summaries. Findings were presented in narrative, tabular, and graphical formats to map the scope and trends in the literature. This includes heatmaps in which characteristics, sampling, and analysis methods of WES were crossmatched with their subsequent public health actions. Identified public health actions that emanated from the included studies were organized in domains. The five most frequently reported public health actions following pathogen detection in wastewater were assessed to determine whether they were implemented individually or in combination with other actions. A thematic division was conducted to categorize the results into two focus areas: 1. public health actions following pathogen detection in wastewater and 2. methods for wastewater sampling and analysis for pathogen detection in wastewater.

## 3 Results

**Figure 1:**
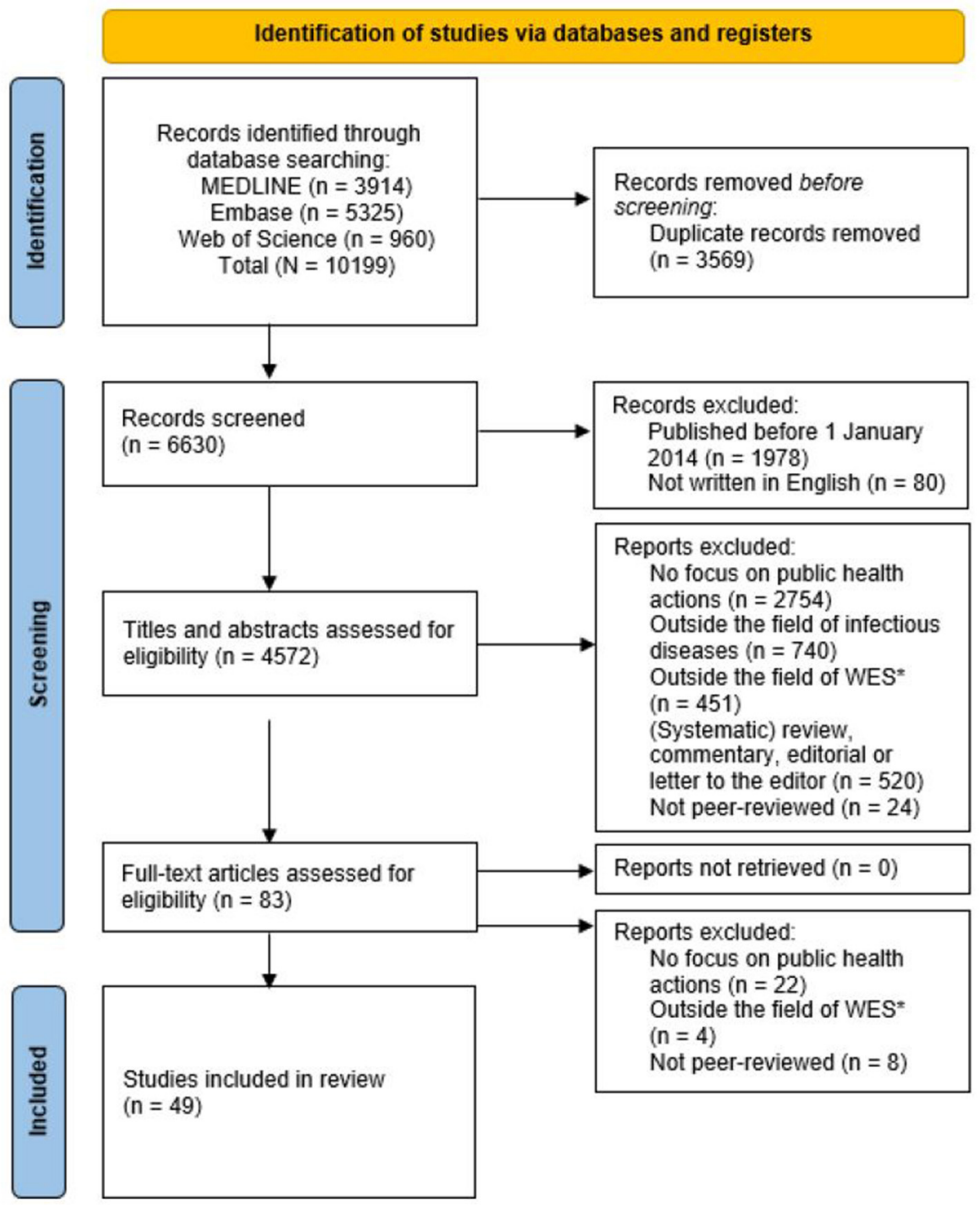
PRISMA flow diagram of the scoping review, 2024.

A total of 10,199 scientific articles were identified through database searching (Figure 1). Out of those, 5,627 were removed (3,569 duplicates and 2,058 records did not meet inclusion criteria). This resulted in 4,572 studies eligible for screening by title and abstract. Of those, 4,489 studies were excluded [no focus on public health actions (*n* = 2,754), outside the field of infectious diseases (*n* = 740), outside the field of WES (*n* = 451), (systematic) review, commentary, editorial or letter to the editor (*n* = 520) or not peer-reviewed (*n* = 24)] resulting in 83 studies being included for full-text review. During full-text review 34 studies were excluded [no focus on public health actions (*n* = 22), outside the field of WES (*n* = 4) or not peer-reviewed (*n* = 8)]. A total of 49 studies were included in the scoping review.

### 3.1 Characteristics of WES surveillance

Table 1 summarizes the characteristics of WES conducted in the included studies (*N* = 49). More detailed information is provided in Supplementary Table 2. Studies were conducted in North America (*n* = 25, 51%) (17, 40–63), Asia (*n* = 11, 22%) (64–74), Europe (*n* = 7, 14%) (75–81), Africa (*n* = 3, 6%) (82–84) and South America (*n* = 3, 6%) (85–87). Most studies were published during or after the early phase of the COVID-19 pandemic between 2020 and 2024 (*n* = 45, 92%). Implementing surveillance (*n* = 30, 61%), tracking of pathogens (*n* = 21, 43%) and outbreak detection (*n* = 16, 33%) were the most described motives for conducting WES. In the majority of conducted WES studies SARS-CoV-2 was the primary pathogen of interest (*n* = 40, 82%) and the most commonly targeted populations were urban residents (*n* = 28, 57%) and on-campus students (*n* = 15, 31%). Most of the WES was commissioned (*n* = 25, 51%) and utilized (*n* = 28, 57%) by local entities and clinical testing data was the most frequently linked data source (*n* = 33, 67%). The majority of studies (*n* = 27, 56%) reported two to four public health actions following WES findings and in 20% (*n* = 10) five or more actions.

**Table 1:** Characteristics and context of wastewater and environmental surveillance (WES) conducted in the included studies in the scoping review on public health actions in response to pathogen detection in wastewater.

**Characteristics and context**	***N* = 49 (%)**
**Study location**	
North America	25 (51%)
Asia	11 (22%)
Europe	7 (14%)
Africa	3 (6%)
South America	3 (6%)
**Publication year**	
2014–2020	4 (8%)
2021–2024	45 (92%)
**Motives for conducted WES** ^*^	
Implement surveillance (e.g., early warning)	30 (61%)
Track pathogen(s) in wastewater (e.g., to monitor trends)	21 (43%)
Identify outbreak(s)	16 (33%)
Inform public health decision-making	11 (22%)
Evaluate interventions (e.g., vaccination campaign)	9 (18%)
Allocate resources	9 (18%)
Monitor variants and mutations	8 (16%)
Complement clinical data	6 (12%)
Detect anomalous pathogen(s) in wastewater	6 (12%)
Assess WES technically	3 (6%)
**Targeted pathogen(s) in WES** ^*^	
SARS-CoV-2^∧^	40 (82%)
Poliovirus	8 (16%)
Influenza virus	1 (2%)
Zika virus	1 (2%)
**Targeted population in WES** ^*^	
Urban residents	28 (57%)
On-campus students	15 (31%)
Rural residents	9 (18%)
Employees working in the same building	5 (10%)
Residents at care facilities	1 (2%)
Not described	1 (2%)
**Data sources linked to WES data** ^*^	
Clinical testing data	33 (67%)
Hospitalization data	6 (12%)
Mortality data	6 (12%)
Syndromic surveillance data	6 (12%)
Immunization data	5 (10%)
Stool sample data	2 (4%)
Not described	8 (16%)
**Entity that commissioned WES** ^*^	
Local (e.g., city council)	25 (51%)
Regional (e.g., state agency)	8 (16%)
National (e.g., Ministry of Health)	16 (33%)
International (e.g., World Health Organization)	–
Not described	5 (10%)
**Entity that utilized WES data** ^*^	
Local (e.g., city council)	28 (57%)
Regional (e.g., state agency)	7 (14%)
National (e.g., Ministry of Health)	19 (39%)
International (e.g., World Health Organization)	1 (2%)
Not described	4 (8%)
**Number of public health actions as response to WES**	
1	12 (24%)
2	13 (27%)
3	7 (14%)
4	7 (14%)
≥5	10 (20%)

^*^Multiple categories per included study. ^∧^SARS-CoV-2, severe acute respiratory syndrome coronavirus 2.

### 3.2 Public health actions

Five thematic domains of public health actions were defined. Reactive and proactive actions for disease control were the most frequently reported domains (both *n* = 30) followed by public health communication and engagement (*n* = 19) (Table 2). In total, 152 public health actions were identified from the included studies comprising a total of 19 specific public health actions. In the domain of reactive actions for disease control (test notifications, isolation or quarantine measures, source and contact tracing, and hygienic measures) were most frequently reported (*n* = 69). This domain aimed at preventing the spread of infectious diseases near the point of origin. Test notifications are defined as targeted messages to inform individuals of potential exposure or risk, encouraging them to get tested for an infectious disease. Frequently reported public health actions in the domain of proactive actions included vaccination and intervention strategies [evaluation of interventions, vaccination activities and behavioral interventions such as enforcing social distancing guidelines (*n* = 33)]. This domain focused on broader public health interventions to reduce disease transmission and impact. Public health actions in the domain of public health communication and community engagement efforts were also frequently reported (*n* = 22). In contrast to the first domain of targeted reactive actions, this domain aimed at community involvement to promote health-protective behaviors and support public cooperation. Less frequently reported public health actions were actions included in the domains of surveillance and monitoring efforts (*n* = 16) and actions focused on policy and collaboration (*n* = 12). For descriptions of all identified public health actions, see Supplementary Table 1 item 28.

**Table 2:** Public health actions in response to pathogen detection in wastewater.

**Included studies**	***N* = 49 (%)**
Reactive actions for disease control	30 (61%)
Test notifications	23 (47%)
Isolation or quarantine measures	15 (31%)
Source tracing	14 (29%)
Contact tracing	11 (22%)
15.6-2.2,-1.3242ptHygienic measures	6 (12%)
Proactive actions for disease control	30 (61%)
Evaluation or adaptation of intervention(s)	15 (31%)
Vaccination campaign initiation, evaluation and/or (re)design	14 (29%)
15.6-2.2,-1.3242ptBehavioural interventions	4 (8%)
Public health communication and engagement	19 (39%)
Public health messaging, promotion or education	19 (39%)
15.6-2.2,-1.3242pt(Increased) community engagement	3 (6%)
Surveillance and monitoring	15 (31%)
Enrichment of epidemiological data	6 (12%)
Implementation of WES^*^ as early warning system	4 (8%)
Expansion of WES	3 (6%)
(Improved) surveillance of (emerging) pathogen(s)	2 (4%)
15.6-2.2,-1.3242ptIncorporation of WES in regular surveillance	1 (2%)
Policy and collaboration	9 (18%)
Initiation or expanded collaboration with external (public health) partners	5 (10%)
Resource allocation	4 (8%)
Public health policy development	2 (4%)
Protect healthcare staff high at risk of severe disease	1 (2%)

Domain counts represent the number of studies reporting at least one action within the domain; action counts represent the total number of times each specific action was reported across all included studies.

^*^WES, wastewater and environmental surveillance.

#### 3.2.1 Joint and solo implementation

**Figure 2:**
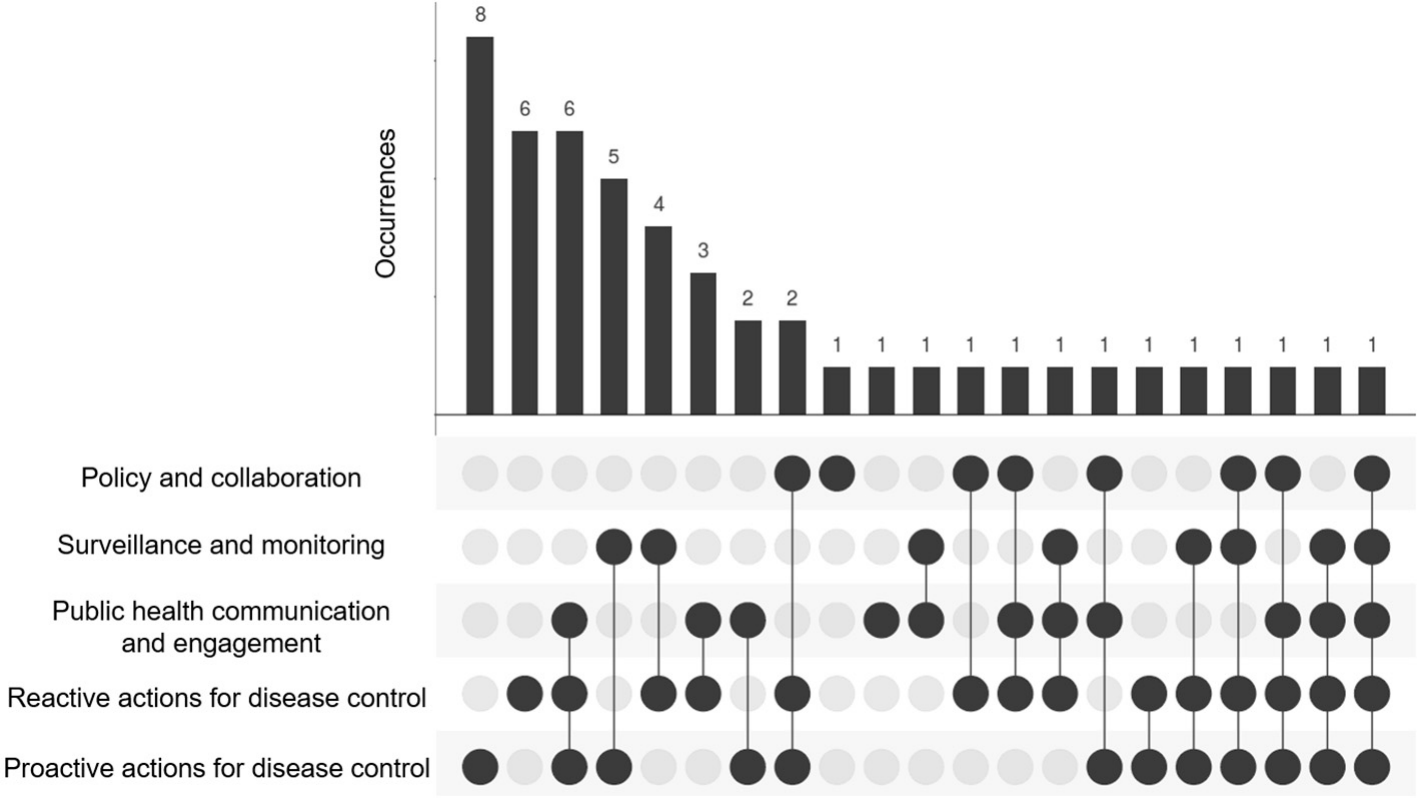
UpSet plot of the five domains of public health actions following pathogen detection in wastewater and their solo and joint implementation with other actions.

Proactive actions for disease control were most often combined with at least one other public health action following wastewater sampling (*n* = 22), see Figure 2. Public health actions in the domain of policy and collaboration were not often combined with other public health actions (*n* = 9). The combined implementation of actions in the domains of public health communication and engagement, reactive actions and proactive actions for disease control were the most frequently reported joint implementation (*n* = 6). Proactive actions for disease control (*n* = 8) and reactive actions for disease control (*n* = 6) were most often reported as standalone actions following wastewater sampling. Standalone actions in the domains of policy and collaboration and public health communication and engagement were each identified once, whereas no standalone actions were identified in the domain of surveillance and monitoring.

#### 3.2.2 Framework for WES

**Figure 3:**
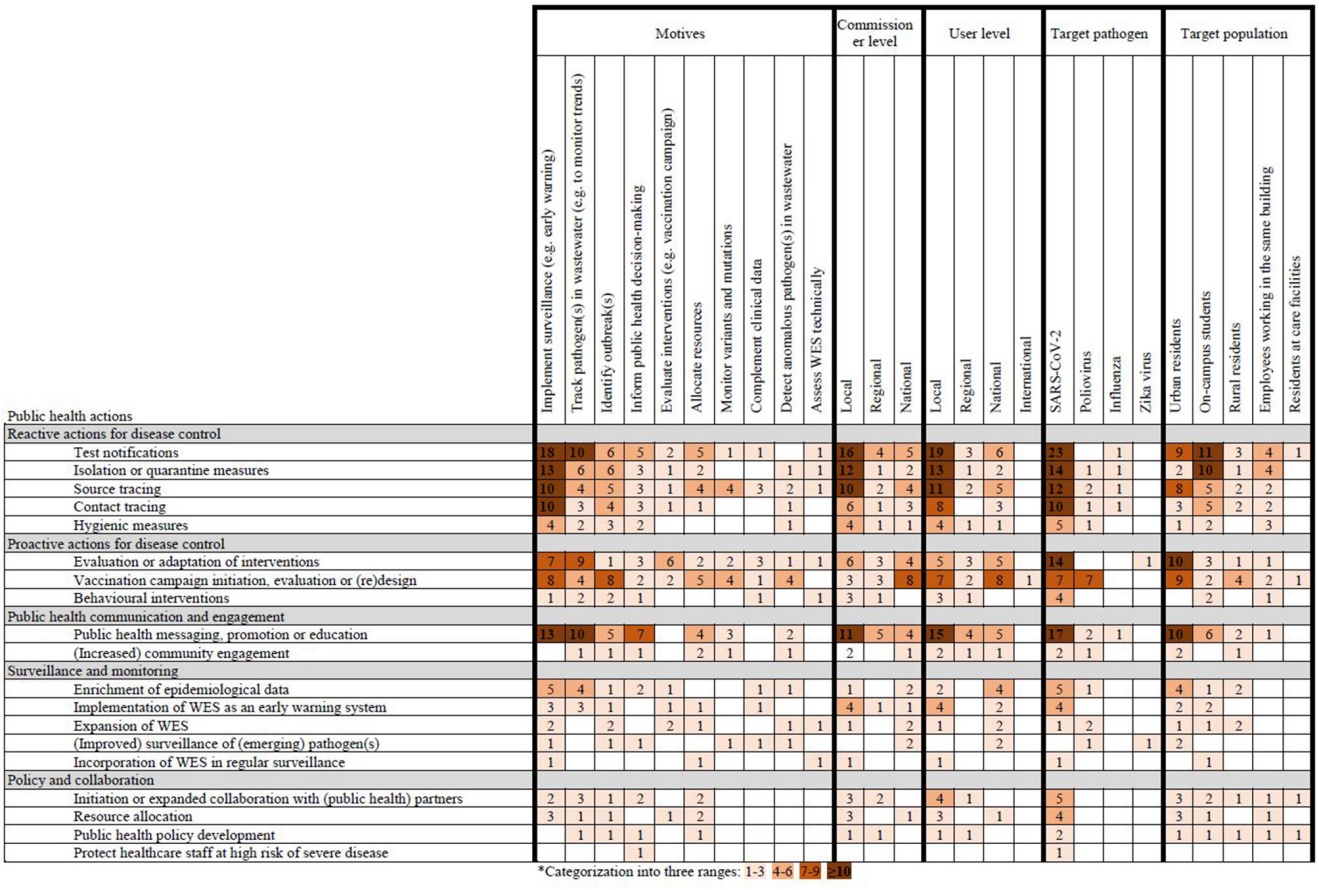
Heatmap of motives, commissioners, users, target pathogens and target populations for wastewater and environmental surveillance (WES) followed up by public health actions*.

For included publications, all motives for WES were at least once followed up by source tracing and evaluation or adaptation of interventions while technical assessment of WES was rarely followed up by a public health action (Figure 3). Surveillance purposes as a motive for WES were most frequently described and followed up by test notifications (*n* = 18), isolation or quarantine measures (*n* = 13), public health messaging, promotion or education (*n* = 13), source tracing (*n* = 10) and contact tracing (*n* = 10). Tracking of pathogens in wastewater as a motive was also frequently described and most often followed up by test notifications (*n* = 10) and public health messaging, promotion or education (*n* = 10). WES locally commissioned and used was most often followed up by reactive public health actions focused on disease control, and public health communication. Public health actions following nationally commissioned WES focused more often on vaccination. Detection of SARS-CoV-2 in wastewater was mostly followed by test notifications (*n* = 23), public health messaging, promotion or education (*n* = 17), isolation or quarantine measures (*n* = 14), evaluation or adaptation of interventions (*n* = 14) and source tracing (*n* = 12). Poliovirus detection in wastewater was mostly linked to vaccination activities (*n* = 7). For on-campus students, public health actions following WES were mostly reactive and focused on disease control while actions for urban residents also focused on proactive actions for disease control.

#### 3.2.3 Wastewater sampling and analysis methods

For included publications (*N* = 49), the most common sampling method was composite sampling (*n* = 33, 67%), followed by grab sampling (*n* = 14, 29%) and passive sampling (*n* = 3, 6%) (Table 3). Wastewater treatment plants and sewer manholes (each *n* = 17, 35%) were the most frequent sampling sites followed by sewer pipes (*n* = 6, 12%). Weekly sampling was more frequently described (*n* = 28, 57%) compared to daily sampling (*n* = 14, 29%). Among the sampling periods, the most frequent identified period spanned more than 300 days (*n* = 20, 41%) followed by those spanning 1–100 days (*n* = 12, 24%). Quantitative detection (*n* = 35, 71%) was the dominant analysis type in comparison with qualitative detection (*n* = 12, 24%) and the majority of analyses used molecular techniques (*n* = 40, 82%) followed by culture-based methods (*n* = 6, 12%). Viral load was the most reported outcome measure (*n* = 27, 55%), followed by binary detection (*n* = 17, 35%).

**Table 3:** Descriptives of wastewater sampling and analysis methods of wastewater and environmental surveillance (WES) conducted in the included studies in the scoping review on public health actions in response to pathogen detection in wastewater.

**Included studies**	***N* = 49 (%)**
**Wastewater sampling technique** ^*^	
Composite sampling	33 (67%)
Grab sampling	14 (29%)
Passive sampling	3 (6%)
Vacuum sampling	1 (2%)
**Wastewater sampling locations**	
Wastewater treatment plant	17 (35%)
Sewer manhole	17 (35%)
Sewer pipe	6 (12%)
Open drains, canals and ditches	4 (8%)
Lift station	2 (4%)
Septic tank	1 (2%)
Sewer ejector pumps	1 (2%)
Sewer pumping station	1 (2%)
**Wastewater sampling frequency** ^*^	
Daily	14 (29%)
Once or more times per week	28 (57%)
Less than once per week	8 (16%)
Not described	2 (4%)
**Wastewater sampling period**	
1–100 days	12 (24%)
101–300 days	11 (22%)
≥301 days	20 (41%)
Not described	6 (12%)
**Wastewater analysis types**	
Quantitative detection	35 (71%)
Qualitative detection	12 (24%)
Not described	3 (6%)
**Wastewater analysis techniques** ^*^	
Molecular	40 (82%)
Culture-based	6 (12%)
Sequencing-based	2 (4%)
Not described	3 (6%)
**Wastewater analysis outcome measures**	
Viral load	27 (55%)
Negative, positive, inconclusive	17 (35%)
Virus type	2 (4%)
Fluorescence	1 (2%)
Not described	2 (4%)

^*^Multiple categories per included study.

**Figure 4:**
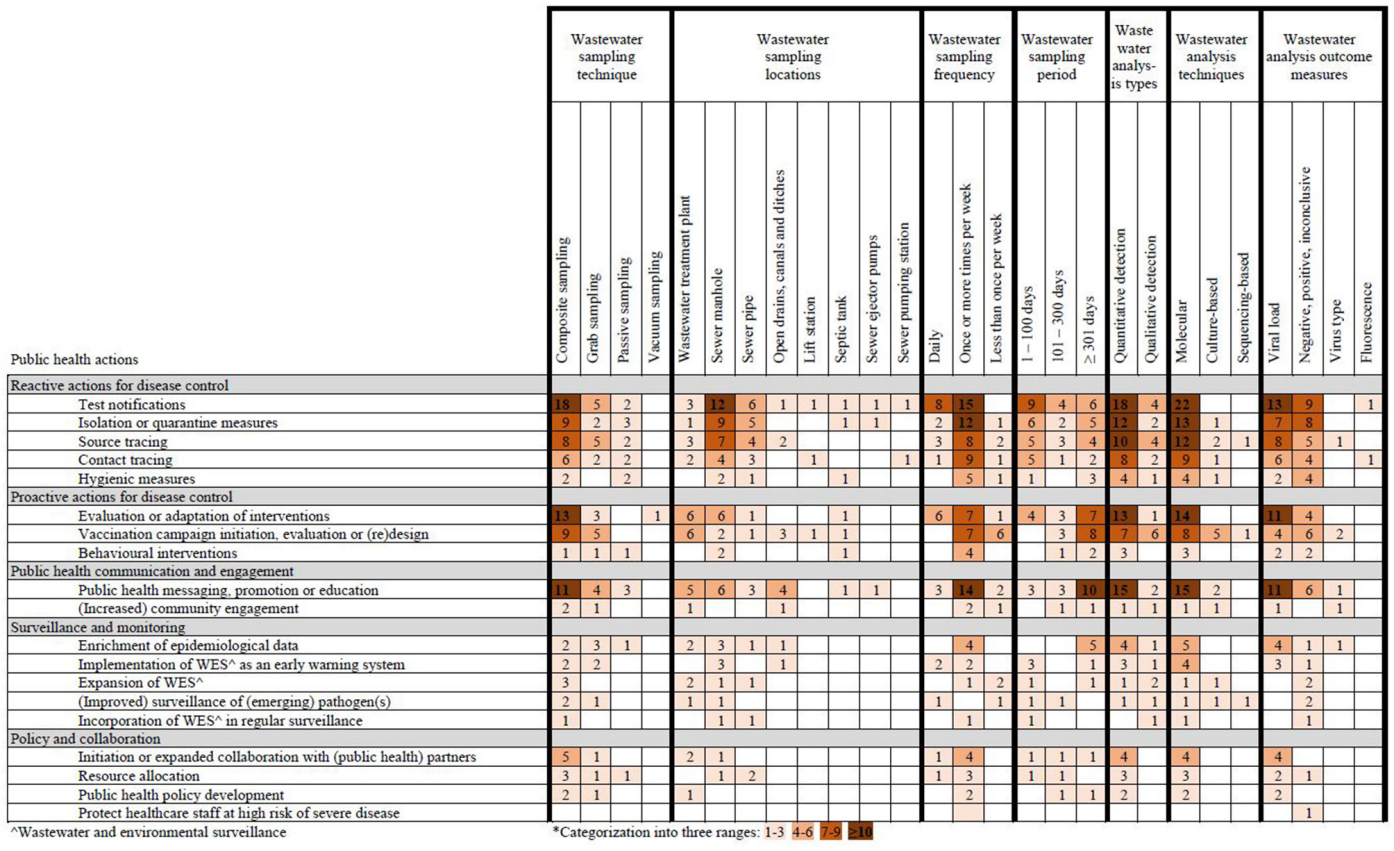
Heatmap of wastewater sampling and analysis methods for pathogen detection in wastewater and their subsequent public health actions*.

All identified wastewater sampling and analysis methods for WES were crossmatched with their subsequent public health actions and are presented in Figure 4. All but one of the identified public health actions were preceded by composite sampling as wastewater sampling technique. Composite sampling was most frequently followed up by test notifications (*n* = 18), evaluation or adaptation of interventions (*n* = 13) and public health messaging, promotion or education (*n* = 11). While wastewater treatment plants were identified as often as sewer manholes as sampling locations, 74% more public health actions were reported in studies using wastewater samples from manholes (61 vs. 35). These actions included test notifications (*n* = 12), isolation or quarantine measures (*n* = 9) and source tracing (*n* = 7). Weekly sampling was followed up mostly by reactive and proactive public health actions focused on disease control, and public health communication. When wastewater sampling was conducted for 1–100 days, public health actions were primarily reactive and focused on disease control. However, when sampling duration was at least 300 days, the emphasis shifted toward public health messaging, vaccination, and intervention strategies. Quantitative and molecular analysis, using viral load as the outcome of wastewater analysis, were most frequently followed up by public health actions. The most common actions for these three analysis elements were test notifications (*n* = 18; *n* = 22; *n* = 13), public health messaging, promotion or education (*n* = 15; *n* = 15; *n* = 11) and evaluation or adaptation of interventions (*n* = 13; *n* = 14; *n* = 11).

## 4 Discussion

To our knowledge, this review is the first to provide an overview of public health actions that were informed by WES and how they were implemented. During the screening phase, the majority of studies were excluded due to the absence of reported public health actions in response to pathogen detection in wastewater. In most cases, public health actions were not integrated into the study design or were presented as speculative or future considerations, nor were stakeholders actively involved in the translation of findings into practice. Most of the included studies were conducted and published in the last 4 years between 2021 and 2024 with urban residents and on-campus students as the most commonly targeted populations. Clinical testing data was most frequently linked as data source to wastewater data and local entities were the most involved in commissioning WES and using the wastewater data. Seventy-five percent of studies reported two or more public health actions following WES and the two most frequently reported public health actions were test notifications and public health messaging, promotion or education.

A strength of this review is that it identifies not only the types of public health actions following wastewater sampling but also their combined applications. Furthermore, our review reflects recent advances in the context of WES and public health integration as we included publications from 2014 onwards. Our study also covered different regions and populations, reflecting global and contextual variation in practice. Another strength is that the review crossmatched wastewater sampling and analysis methods with resulting public health actions, providing insights into methodologies used for each response.

There are also some limitations. First, the language barrier led to the exclusion of papers not written in English, which may partly explain the limited number of studies conducted in non-English speaking and low- and middle-income countries. Second, the review only focused on studies where public health actions followed WES. As a consequence, studies reporting no action or failures to act are underrepresented or excluded, skewing perceptions to an overestimation of impact of public health actions. Third, it is possible that some papers excluded during the title and abstract screening phase for lacking descriptions of public health actions may, in fact, have included those actions within the body of the text. Fourth, it is also likely that some public health actions following WES have not been published in scientific literature, as such dissemination typically occurs only when there is collaboration between researchers and public health authorities. The omission of this grey literature, such as public health bulletins, government guidelines (88) and often peer-reviewed formats such as rapid communications may lead to underreporting of public health actions. These first four limitations have likely introduced systematic bias as highlighted by the exclusion of a substantial number of studies during the screening phase. Future scoping reviews may incorporate Artificial Intelligence-supported tools. This approach could support consistent inclusion of non-English-language studies and grey literature, thereby improving geographic diversity and representation of low- and middle-income countries in the evidence base. However, these approaches should be applied with caution and an awareness of their potential biases and limitations. Fifth, included studies varied widely in design, definitions, sampling methods, and reported outcome. This heterogeneity limited the ability to draw generalizable conclusions or identify best practices. Sixth, the majority of included studies were conducted during the COVID-19 pandemic on SARS-CoV-2 surveillance, limiting insights into broader applications. WES has been conducted for other respiratory viruses such as respiratory syncytial virus (RSV) and influenza (89–91), yet reported public health actions following WES remain scarce. Investigating reasons why WES for these and other infectious diseases does not result into public health interventions warrants further research. However, the pandemic also allowed for validation of sampling and analysis methods and it gave WES a significant boost, expanding its potential for other pathogens. Seventh, as we excluded literature published prior to 2014, pioneering studies on WES conducted as part of polio eradication programs were not included in our analysis (92). Last, most included studies were conducted in high-resource settings, limiting the applicability of findings to global public health settings, particularly in regions with limited infrastructure for WES.

To our knowledge, there is only one other systematic review that looked at public health actions following WES. Kilaru et al. identified several studies conducted pre-COVID that described public health interventions following WES (33). These studies focused solely on vaccination campaign guidance and evaluation for poliovirus, whereas our review also identified non-COVID studies describing public health actions related to disease control, health communication, and surveillance.

We identified a surge in studies from 2021 to 2024 primarily targeting urban residents and on-campus students during the COVID-19 pandemic. This indicates that WES leading to public health actions is a recent development deployed in high-density areas. While surveillance purposes were most often described as motive for WES, the majority of described public health actions were reactive rather than proactive, focusing on disease control rather than surveillance and monitoring efforts. This suggests that WES holds unexplored potential as a proactive tool for guiding preventive public health actions. SARS-CoV-2 detection in wastewater triggered a broad range of public health responses, while wastewater-based poliovirus surveillance primarily led to vaccination efforts. Different target populations received tailored public health actions, reflecting the need for distinct approaches based on the characteristics and needs of each population. Frequent linking of wastewater and clinical data demonstrated the role of WES in complementing traditional surveillance and diagnostic methods.

The most commonly implemented wastewater sampling and analysis methods were frequently followed up by public health actions with a focus on disease control, or specific actions such as public health messaging and evaluation of interventions. This indicates that WES conducted at a local scale has a great potential to inform public health responses by serving as a tool for early detection, monitoring, and response to public health threats, enabling timely interventions to mitigate disease spread in specific communities. However, causal relationship between WES and reported public health actions is debatable, as the same measures could have been taken in the absence of WES. In some of the included studies, it was unclear what role other surveillance tools played in prompting public health actions following pathogen detection in wastewater, highlighting the need for future studies to clearly specify the contribution of each surveillance tool. Furthermore, each future WES study should include proactive governance and stakeholder engagement (11, 12). Also, finding a balance between the potential high resolution of WES and the implementation of targeted public health responses remains a topic of debate because of ethical, privacy and legal implications (93, 94).

We found that national-level WES focused more on actions with respect to vaccination as such activities are often conducted on a national scale, while local-level efforts prioritized immediate containment. Most WES was commissioned and used by local entities and sewer manholes were sampled as often as wastewater treatment plants which emphasizes the interest and application of local WES by these entities. Although long-term WES (≥301 days) was reported most frequently it was rarely followed up by public health actions focusing on improved long-term surveillance and monitoring. It is possible that many wastewater surveillance programs were initially implemented for short-term outbreak response, but continued due to cost-effectiveness, infrastructure investments, and the growing recognition of wastewater data as a valuable tool for epidemiological surveillance (95, 96).

Though WES exists for several decades (97), SARS-CoV-2 is the first target that is quantified by qRT-PCR (98). This aligns with our findings, which show that quantitative detection methods were used mainly for SARS-CoV-2, while qualitative or culture-based approaches were predominantly applied to polio and other pathogens. More than half of the studies measured viral load rather than binary detection (positive/negative). This can be explained by the need for quantitative data for sufficient interpretation of wastewater signals to conduct public health actions, as it allows for tracking trends, estimating infection prevalence and linking wastewater dynamics to epidemiological trends. Only a few of the included studies based the reported public health actions on data obtained through sequencing-based methodologies. Although sequencing could provide valuable insights, such as tracking the emergence and spread of variants (99), public health actions based on these data have so far been limited.

This scoping review shows that WES is not just a passive monitoring tool: it has actively guided a wide range of public health actions. It provides the first mapped evidence on how various wastewater sampling and analysis methods were translated into specific actions, helping public health professionals make evidence-based decisions. Most included studies focused on SARS-CoV-2 and were conducted in high-income regions. Further studies on WES focusing on other pandemic-prone, vector-borne zoonotic, and high-burden infectious diseases such as dengue, Zika and West Nile that predominantly occur in low-resource settings, are necessary. Also, studies conducted in low- and middle-income settings are needed to allow more efficient allocation of potentially limited resources in these contexts. Rural residents, building employees, and residents in care facilities were less frequently targeted, which may indicate gaps in monitoring underserved or vulnerable populations. The variability in conducted public health actions and lack of long-term integration of WES highlight areas for further research.

## Data Availability

The datasets generated and analyzed during the current study will become available in the Dataverse repository (100).
