# Supplementary material for: Public health actions in response to pathogen detection in wastewater and the environment: a scoping review
Source: Front Public Health. 2026 Jan 16;13:1675742. doi: 10.3389/fpubh.2025.1675742 (PMC12855521; doi:10.3389/fpubh.2025.1675742)
Supplement: Supplementary file 1 [file Data_Sheet_1.pdf]

Supplementary Material to

Public health actions in response to pathogen detection in wastewater and the environment: a scoping review

Maarten de Jong[1,6]*, Jolinda de Korne-Elenbaas[2], Ewout Fanoy[3], Gertjan Medema[4], Miranda de Graaf[5], Maria Prins[1,6], Maarten F. Schim van der Loeff[1,6], Joost Daams[7], Ana Maria de Roda Husman[8,9], Janneke C.M. Heijne[1,6]

Supplementary Data Sheet 1: Preferred Reporting Items for Systematic reviews and Meta-Analyses extension for Scoping Reviews (PRISMA-ScR)-checklist

SECTION	ITEM	PRISMA-ScR CHECKLIST ITEM	REPORTED ON PAGE #
**TITLE**			
Title	1	Identify the report as a scoping review.	1
**ABSTRACT**			
Structured summary	2	Provide a structured summary that includes (as applicable): background, objectives, eligibility criteria, sources of evidence, charting methods, results, and conclusions that relate to the review questions and objectives.	3-4
**INTRODUCTION**			
Rationale	3	Describe the rationale for the review in the context of what is already known. Explain why the review questions/objectives lend themselves to a scoping review approach.	5
Objectives	4	Provide an explicit statement of the questions and objectives being addressed with reference to their key elements (e.g., population or participants, concepts, and context) or other relevant key elements used to conceptualize the review questions and/or objectives.	6
**METHODS**			
Protocol and registration	5	Indicate whether a review protocol exists; state if and where it can be accessed (e.g., a Web address); and if available, provide registration information, including the registration number.	6

SECTION	ITEM	PRISMA-ScR CHECKLIST ITEM	REPORTED ON PAGE #
Eligibility criteria	6	Specify characteristics of the sources of evidence used as eligibility criteria (e.g., years considered, language, and publication status), and provide a rationale.	7
Information sources*	7	Describe all information sources in the search (e.g., databases with dates of coverage and contact with authors to identify additional sources), as well as the date the most recent search was executed.	7
Search	8	Present the full electronic search strategy for at least 1 database, including any limits used, such that it could be repeated.	7
Selection of sources of evidence†	9	State the process for selecting sources of evidence (i.e., screening and eligibility) included in the scoping review.	7-8
Data charting process‡	10	Describe the methods of charting data from the included sources of evidence (e.g., calibrated forms or forms that have been tested by the team before their use, and whether data charting was done independently or in duplicate) and any processes for obtaining and confirming data from investigators.	8
Data items	11	List and define all variables for which data were sought and any assumptions and simplifications made.	8
Critical appraisal of individual sources of evidence§	12	If done, provide a rationale for conducting a critical appraisal of included sources of evidence; describe the methods used and how this information was used in any data synthesis (if appropriate).	/
Synthesis of results	13	Describe the methods of handling and summarizing the data that were charted.	8-9
**RESULTS**			

SECTION	ITEM	PRISMA-ScR CHECKLIST ITEM	REPORTED ON PAGE #
Selection of sources of evidence	14	Give numbers of sources of evidence screened, assessed for eligibility, and included in the review, with reasons for exclusions at each stage, ideally using a flow diagram.	9
Characteristics of sources of evidence	15	For each source of evidence, present characteristics for which data were charted and provide the citations.	9
Critical appraisal within sources of evidence	16	If done, present data on critical appraisal of included sources of evidence (see item 12).	/
Results of individual sources of evidence	17	For each included source of evidence, present the relevant data that were charted that relate to the review questions and objectives.	9
Synthesis of results	18	Summarize and/or present the charting results as they relate to the review questions and objectives.	10-13
**DISCUSSION**			
Summary of evidence	19	Summarize the main results (including an overview of concepts, themes, and types of evidence available), link to the review questions and objectives, and consider the relevance to key groups.	13-16
Limitations	20	Discuss the limitations of the scoping review process.	14
Conclusions	21	Provide a general interpretation of the results with respect to the review questions and objectives, as well as potential implications and/or next steps.	17
**FUNDING**			
Funding	22	Describe sources of funding for the included sources of evidence, as well as sources of funding	18

SECTION	ITEM	PRISMA-ScR CHECKLIST ITEM	REPORTED ON PAGE #
		for the scoping review. Describe the role of the funders of the scoping review.	

Supplementary Data Sheet 2: Detailed search string

Ovid MEDLINE(R) ALL <1946 to July 25, 2024>
Search date: 26 July 2024

#	Searches	Results
1	Sewage/ch, is, ip, mt, mi, ps, pc, vi	19530
2	(wastewater/ or sewage/ or water pollution/) and (exp communicable disease control/ or epidemiological monitoring/ or exp environmental monitoring/)	42459
3	(wastewater/ or sewage/ or water pollution/) and isolation purification.fs.	12535
4	Wastewater-Based Epidemiological Monitoring/	652
5	((wastewater? or sewage) adj3 (sampl* or surveil* or monitor* or treatment plant? or test*)).ab,kf,ti.	24183
6	((wastewater? or sewage) adj10 (detect* or charact* or concentration)).ab,kf,ti.	13100
7	wastewater microbiome.ab,kf,ti.	20
8	sewage signal*.ab,kf,ti.	8
9	or/1-8 [sewage sampling]	72752
10	communicable disease control/ or public health/	129789
11	epidemiology.fs.	2225372
12	(public health or lockdown or virus spread or epidemiol* or epidemic* or endemic* or pandemic* or community wastewater? or ((spread or surveillance) adj5 (infection or disease or virus* or norovirus* or bacter* or parasit* or fung*)) or (spread adj3 infection)).ab,kf,ti.	1332877
13	or/10-12 [public health]	3089811
14	communicable diseases/	34862
15	(communicable disease? or fungi or bacter* or parasite? or virus* or viral or norovirus or infect*).ab,kf,ti.	3676394
16	9 and 13	5834
17	limit 16 to covid-19	1550
18	or/14-15,17 [infectious agents]	3684127
19	and/9,13,18	3745
20	wastewater based epidemiological monitoring/	652
21	19 or 20	3914

Ovid Embase Classic+Embase <1947 to 2024 July 25>
Search date: 26 July 2024

#	Searches	Results
1	Sewage/	26253
2	(exp wastewater/ or sewage/ or exp water pollution/) and (exp communicable disease control/ or exp epidemiological monitoring/ or environmental monitoring/ or exp environmental surveillance/ or water monitoring/)	21796
3	Wastewater-Based Epidemiology/	1263
4	((wastewater? or sewage) adj3 (sampl* or surveil* or monitor* or treatment plant? or test*)).ab,kw,ti.	28829
5	((wastewater? or sewage) adj10 (detect* or charact* or concentration)).ab,kw,ti.	15300
6	wastewater microbiome.ab,kw,ti.	22
7	sewage signal*.ab,kw,ti.	7
8	or/1-7 [sewage sampling]	77211
9	exp communicable disease control/ or public health/	428673
10	epidemiology.fs.	1267010
11	(public health or lockdown or virus spread or epidemiol* or epidemic* or endemic* or pandemic* or community wastewater? or ((spread or surveillance) adj5 (infection or disease or virus* or norovirus* or bacter* or parasit* or fung*)) or (spread adj3 infection)).ab,kw,ti.	1620542
12	or/9-11 [public health]	2740353
13	exp *infection/ or *fungus/ or *parasite/ or *virus/	3025424
14	(communicable disease? or fungi or bacter* or parasite? or virus* or viral or norovirus or infect*).ab,kw,ti.	4654106
15	8 and 12	6971
16	limit 15 to covid-19	1642
17	or/13-14,16 [infectious agents]	5988293
18	and/8,12,17	4706
19	wastewater based epidemiology.mp.	1758
20	18 or 19	5325

Web of Science
Search date: 26 July 2024

#	Searches	Results
1	TS=((wastewater? or sewage) NEAR/2 (sampl* or surveil* or monitor* or "treatment plant?" or test*))	8873
2	TS=((wastewater? or sewage) NEAR/9 (detect* or charact* or concentration))	9457
3	TS="wastewater microbiome"	24
4	TS="sewage signal*"	10
5	#1 OR #2 OR #3 OR #4	16576
6	TS=("public health" or lockdown or virus spread or epidemiol* or epidemic* or endemic* or pandemic* or "community wastewater?" or ((spread or surveillance) NEAR/4 (infection or disease or virus* or norovirus* or bacter* or parasit* or fung*)) or (spread NEAR/2 infection))	1685300
7	TS=("communicable disease?" or fungi or bacter* or parasite? or virus* or viral or norovirus or infect*)	4394083
8	#5 AND #6 AND #7	960

Supplementary Table 1: Data Extraction Table

**Study specifics**		
1.	Study title	*Title of the study*
2.	First author(s)	*Name of first author(s)*
3.	Other author(s)	*Name of co-author(s)*
4.	Publication year	*Year of publication*
5.	Journal	*Journal of publication*
6.	Calendar year(s) of data collection	*Year(s) in which the described data were collected*
7.	Geographical context	*a. Country in which the study data were collected*<br>*b. Region or province or city*<br>*c. Setting in which the study data were collected*
**Study context**		
8.	Aim(s) description	*Copy or briefly describe the aim(s) of the conducted study*
9.	Motive	*Specify the motive or reason for the conducted sewage surveillance:*<br>*(a) Detect anomalous pathogen(s) in sewage*<br>*(b) Track pathogen(s) in sewage (e.g. to monitor trends)*<br>*(c) Monitor variants and mutations*<br>*(d) Identify outbreak(s)*<br>*(e) Surveillance purposes (e.g. early warning)*<br>*(f) Evaluate interventions (e.g. vaccination campaign)*<br>*(g) Resource allocation*<br>*(h) Complementing clinical data*

	(i) Otherwise: …<br><br>*(j) N/A*
10. Targeted pathogen(s)	*a. Number of pathogens the study focused on*<br><br>*b. Pathogen(s) the study focused on*
11. Target population	*Briefly describe the study population (e.g. people living in a specific region, attending a school, residing in a hospital, etc.)*
12. Clinical data	*If the study compares clinical data with sewage data, briefly describe clinical setting, sampling regime, type of samples, etc.*
13. Commissioner	*Briefly describe the entity (local, regional, national) that instructed the sewage surveillance*<br><br>*Local entities: municipal or community-level organizations, such as city health departments, local wastewater utilities, or town councils, that operate within a specific locality or neighborhood*<br><br>*Regional entities: oversee multiple local jurisdictions within a broader geographic area, such as a state, province, or health district, and may coordinate efforts across municipalities*<br><br>*National entities: operate at the country level and are responsible for setting overarching policies, regulations, and strategic public health responses—examples include national public health institutes or federal health ministries*
14. End user	*Briefly describe the entity (local, regional, national) that utilized the outcome(s) of the sewage surveillance*
**Sewage sampling methods**	
15. Sampling naming	*Copy or briefly describe the naming of the sewage sampling method(s)*
16. Sampling method(s)	*Specify if the sampling method was*<br><br>*(a) grab sampling*

	(b) composite sampling<br>*(c) automatic sampling*<br>*(d) passive sampling*<br>*(e) otherwise: …*<br>*(f) N/A*
17. Sampling location(s)	*Specify the sewage sampling site(s):*<br>*(a) Sewer manhole*<br>*(b) Sewer pipe*<br>*(c) Sewer pumping station*<br>*(d) Wastewater treatment plant*<br>*(e) Non-sewered setting: …*<br>*(f) Otherwise: …*<br>*(g) N/A*
18. Sampling technique	*Briefly describe the:*<br>*a. sample frequency*<br>*b. quantity of each collected sample (in mL)*<br>*c. period of time between first and last sample taken (in days)*
**Sewage analysis methods**	
19. Analysis naming	*Copy or briefly describe the naming of the sewage analysis method(s)*
20. Type of analysis	*a. Qualitative (detection yes/no)*<br>*b. Quantitative (concentrations, relative abundances, etc.)*<br>*c. Otherwise: …*
21. Analysis technique(s)	*Specify if the analysis method was:*

		(a) culture-based (e.g. bacterial or viral or otherwise: ...)<br><br>*(b) molecular (e.g. qPCR*, dPCR*, LAMP* or otherwise: ...)*<br><br>*(c) sequencing-based (e.g. Sanger, Illumina short-read, Nanopore long-read, amplicon-based or otherwise ...)*<br><br>*(d) metagenomics-based (e.g. 16S, viral metagenomics or otherwise: ...)*<br><br>*(e) otherwise: ...*<br><br>*(f) N/A*
22.	Analysis target	*Specify whether the target is live bacteria or replication competent virus (in case of culture), a specific gene (in case of molecular), the whole genome, 16s, etc.*
23.	Sample processing methodology	*Specify if the method for sample concentration and nucleic acid extraction was:*<br><br>*(a) ultrafiltration (size-based)*<br><br>*(b) electronegative membrane filtration*<br><br>*(c) absorption-precipitation (PEG precipitation)*<br><br>*(d) flocculation - centrifugation*<br><br>*(e) filter-based direct capture*<br><br>*(f) magnetic bead-based methods*<br><br>*(g) otherwise: ...*<br><br>*(h) N/A*
24.	Data normalization	*Specify if the applied data normalization was:*<br><br>*(a) PMMoV**<br><br>*(b) CrAssphage**<br><br>*(c) flow normalization*<br><br>*(d) otherwise: ...*

	(e) N/A
25. Analysis protocol(s)	*Briefly describe the applied analysis protocol(s) applied on the sewage samples*
26. Outcome measure(s)	*Briefly describe the outcome measure(s) of the analysis method(s) (e.g. concentrations in units)*
**Public health actions**	
27. Description public health action(s)	*Copy or briefly describe the public health action(s) as a response to the conducted sewage surveillance*
28. Public health action(s) as a response to the conducted sewage surveillance	*Specify if the conducted sewage surveillance led to:*<br><br>*(a) isolation or quarantine measures*<br>*(separating or restricting movements of individuals)*<br><br>*(b) hygienic measures*<br>*(practices aimed at maintaining cleanliness and preventing the spread of disease, such as handwashing, surface disinfection, etc.)*<br><br>*(c) source tracing*<br>*(identifying the origin and transmission pathways of an infectious disease)*<br><br>*(d) contact tracing*<br>*(identifying, assessing, and monitoring people who have been exposed to an infectious disease)*<br><br>*(e) test notifications*<br>*(messages to inform individuals of potential exposure or risk, encouraging them to get tested for an infectious disease)*<br><br>*(f) vaccination campaign initiation, evaluation and/or (re)design*<br>*(launching immunization efforts, assessing their effectiveness and reach, and adjusting strategies)*<br><br>*(g) public health messaging, promotion and/or education*<br>*(developing and delivering information to raise awareness, influence behavior, and empower communities to make informed health decisions)*

	(h) (increased) community engagement<br>*(actively involving local populations in public health planning and response efforts to build trust and enhance the effectiveness of interventions)*<br><br>*(i) behavioral interventions*<br>*(influence individuals' actions and habits to promote behaviors that reduce the spread or impact of disease)*<br><br>*(j) vector control measures*<br>*(reducing or eliminating disease-carrying organisms, such as mosquitoes or rodents)*<br><br>*(k) incorporation of sewage surveillance in outbreak protocol(s)*<br>*(integrating sewage surveillance data into existing public health response plans)*<br><br>*(l) incorporation of sewage surveillance in regular surveillance*<br>*(using sewage data as a routine tool to monitor population-level health trends)*<br><br>*(m) (improved) surveillance of (emerging) pathogen(s)*<br>*(enhancing systems to detect, monitor, and analyze new or re-emerging infectious agents)*<br><br>*(n) enrichment of epidemiological data*<br>*(use sewage surveillance to enhance the accuracy, depth, and usefulness of disease surveillance)*<br><br>*(o) implementation of sewage surveillance as early warning system*<br>*(using sewage analysis to detect signs of infectious disease circulation in a population before clinical cases rise)*<br><br>*(p) evaluation and/or adaptation of existing intervention(s)*<br>*(assessing the effectiveness of current public health measures and making necessary adjustments)*<br><br>*(q) enhanced (pandemic) preparedness for future outbreaks*<br>*(strengthening systems, resources, and strategies to improve the ability to detect, respond to, and manage future infectious disease threats)*<br><br>*(r) new research (in the field of public health)*<br>*(sewage surveillance findings reveal knowledge gaps or emerging trends that prompt further investigation*

	(s) public health policy development<br>*(action when surveillance data highlight risks, trends, or gaps that necessitate the creation or revision of policies)*<br><br>*(t) initiation or expanded collaboration with (public health) partners*<br>*(findings indicate a need for coordinated efforts, resource sharing, or joint responses)*<br><br>*(u) otherwise: …*
29. Accomplishment	*Briefly describe whether the public health action contributed to the intended objective of the sewage surveillance*
30. Recommendation(s)	*Copy or briefly describe the recommendation(s) for public health action(s) of the sewage surveillance*

*qPCR: quantitative polymerase chain reaction, dPCR: digital polymerase chain reaction, LAMP: loop-mediated isothermal amplification, PEG: polyethylene glycol, PMMoV: pepper mild mottle virus, CrAssphage: a broad group of diverse bacteriophages in the order Caudovirales

Supplementary Table 2: Included studies in the scoping review on public health actions in response to pathogen detection in wastewater

First author and reference	Publication year	Country of study	Target pathogen(s)	Target population	Data sources linked to wastewater data	Commissioner	End user	Public health actions as response to wastewater and environmental surveillance*
Akingbola et al. (33)	2022	Canada	SARS-CoV-2	Shelter residents and on-site staff	COVID-19 positive tests	Toronto Metropolitan University	Toronto Public Health and the shelter	a, b, c, d, e, u
Alonso et al. (34)	2023	Unites States of America	SARS-CoV-2	Citizens of Chelsea	Vaccination rate data	The Chelsea Project: a collaborative effort in which government entities, local nonprofit organizations, and startups	Local community-based organizations (CBO), the Chelsea Department of Public Health, public health directors from two CBOs and the city's communications director	f, g, h, t, u
Betancourt et al. (35)	2021	United States of America	SARS-CoV-2	Students residing at University of Arizona	COVID-19 positive tests	University of Arizona (Wastewater-based epidemiology Expert Team, as part of the Task Force and Campus Re-Entry Working Groups)	University of Arizona (Wastewater-based epidemiology Expert Team)	a, c, d, e, o
Bohrerova et al. (36)	2023	United States of America	SARS-CoV-2	Ohio citizens	Not described	Ohio Department of Health and the Ohio Environmental Protection Agency	Local public health officials	d, e, f, g, t

Bowes et al. (37)	2023	United States of America	SARS-CoV-2	Citizens of Tempe and Guadalupe	Data on new positive cases, COVID-19-related hospitalizations, deaths, and long-term care facility deaths per day	Tempe and Arizona State University	Not described	g, p
Brooks et al. (38)	2023	United States of America	SARS-CoV-2^	Community of Yarmouth	Data of weekly reported COVID-19 cases	Yarmouth Wastewater Testing Team	Town manager and residents, Yarmouth Community Coronavirus Task Force (YCCTF), Yarmouth Wastewater Testing Team and Maine Department of Health and Human Services	g, h
Chaudhuri et al. (58)	2023	India	SARS-CoV-2	Population of Bengaluru	Weekly clinical positivity data	Government of Karnataka and Bruhat Bengaluru Mahanagara Palike	Local public health bodies, general populace, clinicians, members of the scientific community, journalists reporting on COVID-19, office-administrators and office-goers for their diverse purposes	g, o
Cheng et al. (39)	2023	Canada	SARS-CoV-2	Residents of Regional Municipality of Peel	COVID-19 patient, hospitalization and testing data	Regional Municipality of Peel	Peel Public Health	s, t

Corchis-Scott et al. (39)	2023	Canada	SARS-CoV-2	Students residing at three resident halls in Windsor University	Data of COVID-19 cases in the Windsor-Essex region	University of Windsor	University of Windsor	a, b, d, e, g, i
Corchis-Scott et al. (40)	2021	Canada	SARS-CoV-2	Residents of Windsor University	Not described	University of Windsor	University of Windsor	a, c, d, e, g, u,
Daigle et al. (42)	2022	Canada	SARS-CoV-2	Remote community of Yellowknife	Not described	Public Health Agency of Canada-National Microbiology Laboratory, Winnipeg	Office of the Chief Public Health Officer	d, e
Deng et al. (59)	2022	China	SARS-CoV-2	33,000 people in Tai Po district	Clinical data related to the confirmed case	Collaboration the University of Hong Kong and the Hongkong Government	The University of Hong Kong and the Hongkong Government	c, e
Deng et al. (60)	2022	China	SARS-CoV-2	Hongkong citizens	Data of confirmed COVID-19 patients in Hong Kong, including report dates, hospital admission dates, and discharge dates	Hongkong governmental bodies and University of Hongkong	Hongkong governmental bodies and University of Hongkong	c, e, g, p
Duizer et al. (69)	2023	The Netherlands	Poliovirus	Employees of poliovirus facilities	Not described	National authority for containment of poliovirus	National authority for containment of poliovirus	a, b, d

Faye et al. (76)	2022	Senegal	Poliovirus	People living in and around the city of Dakar	Stool samples from acute flaccid paralysis cases were collected	Collaboration between the Prevention Office at the Senegalese Ministry of Health and Social Action, the Dakar Medical Region, Institut Pasteur de Dakar, the National Office of Sanitization in Senegal, and World Health Organization	Ministry of Health and Social Action and the World Health Organization country and African region offices	f
Gibas et al. (43)	2021	United States of America	SARS-CoV-2	Residents at University UNC Charlotte Campus	Not described	UNC Charlotte	UNC Charlotte	a, c, e, l, v
Harris-Lovett et al. (44)	2023	United States of America	SARS-CoV-2	Respondents responsible for public health decision making	Not described	Not described	Not described	a, b, d, e, g, i, n, p, w
Haskell et al. (45)	2023	Canada	SARS-CoV-2	Residents at Waterloo University campus	Clinical case data based on students self-reporting illness and self-reported test results	University of Waterloo	University of Waterloo	a, b, c, g, n
Hillary et al. (70)	2021	United Kingdom	SARS-CoV-2	Inhabitants of Wales and Northwest England	COVID-19 positive tests and deaths	English government	English government, and local city councils	p

Innes et al. (46)	2022	United States of America	SARS-CoV-2	Workers at food processing facility	Reported COVID-19 case counts in the food processor facility’s surrounding community	University of Arizona Yuma Center of Excellence for Desert Agriculture in coordination with Yuma County Public Health Services District, the Regional Center for Border Health, Inc., and the Arizona Department of Health Services	University of Arizona Yuma Center of Excellence for Desert Agriculture in coordination with Yuma County Public Health Services District, the Regional Center for Border Health, Inc., and the Arizona Department of Health Services	a, b, e, f, g, i, p
Johnson Muluh et al. (77)	2016	Nigeria	Poliovirus	High risk populations in Nigerian states	Acute Flaccid Paralysis surveillance was performed simultaneously	Federal Ministry of Health, the National Primary Health Care Development Agency and state ministries of environment and health	National Polio Emergency Operations Center	c, f, g, h
Karthikeyan et al. (47)	2021	Unites States of America	SARS-CoV-2	Students at San Diego University	COVID-19 positive cases	University of California San Diego	University of California San Diego	a, e, g
Kisand et al. (71)	2023	Estonia	SARS-CoV-2	Larger towns and smaller towns	COVID-19 positive cases	Not described	Estonian Health Board	e, n
Klapsa et al. (72)	2022	United Kingdom	Poliovirus	Citizens of London	Not described	UK Health Security Agency	UK Health Security Agency	f, m
Lin et al. (48)	2023	Unites States of America	SARS-CoV-2	Residents University of California San Diego	COVID-19 test results	University of California San Diego	University of California San Diego	e, g
Manor et al. (61)	2014	Israel	Poliovirus	70% of Israeli population	Not described	Public Health Services of the Ministry of Health	Public Health Services of the Ministry of Health	f, v

Morais et al. (78)	2023	Angola	Poliovirus	Inhabitants of selected cities and provinces	Data on acute flaccid paralysis surveillance, reported vaccine derived poliovirus type 2 cases and supplementary immunization activities	Angolan government	Angolan Ministry of Health and World Health Organization	f, n
Ng et al. (62)	2023	China	SARS-CoV-2	Citizens of Hongkong	Confirmed COVID-19 cases	Hong Kong Special Administrative Region Government	Hong Kong Special Administrative Region Government	e, p, u
Pardo-Figueroa et al. (79)	2022	Peru	SARS-CoV-2	Citizens of Lima, Callao and Arequipa	COVID-19 mortality, incidence, occupancy of intensive care unit beds, occupancy of hospital and vaccination status	Not described	Peruvian Health Authorities	c, n, p
Porter et al. (49)	2024	United States of America	SARS-CoV-2	Students at Grand Valley State University and Oakland University	COVID-19 Clinical case data	First by State of Michigan, later locally overtaken by each university	Grand Valley State University and Oakland University	p
Prado et al. (80)	2021	Brasil	SARS-CoV-2	Citizens of Niterói municipality	Confirmed COVID-19 cases and deaths	Niterói municipality with Oswaldo Cruz Foundation – Fiocruz	Niterói municipality with Oswaldo Cruz Foundation – Fiocruz	a, c, d, e, g
Reeves et al. (50)	2021	United States of	SARS-CoV-2	Residents of Colorado	Weekly individualized saliva-monitoring	Colorado University	Colorado University	a, d, e, o, p, t

		America		University Campus	RT-qPCR^ testing of all asymptomatic on-campus residents			
Rodríguez et al. (81)	2023	Guatemala	Poliovirus	Citizens of Villa Nueva and San Juan Sacatepéquez	Acute flaccid paralysis incidences reported by hospitals	Guatemala Ministry of Health	Guatemala Ministry of Health	c, f, g
Rouchka et al. (51)	2021	United States of America	SARS-CoV-2	Citizens of Louisville	Confirmed COVID-19 cases	Public health and metropolitan wastewater management authorities and the University of Louisville	Public health and metropolitan wastewater management authorities and the University of Louisville	f, g
Scott et al. (52)	2021	United States of America	SARS-CoV-2	Tulane university students residing at university campus	Nasal swab test results of all Tulane University students, faculty and staff	Tulane University	Tulane University	i
Sharaby et al. (63)	2023	Israel	SARS-CoV-2	Residents and staff at Technion University Campus	Reported COVID-19-related information, such as clinical test result and date, and quarantine start and end dates	Technion University Campus	Technion University Campus	a, c, e
Stephens et al. (73)	2022	The Netherlands	SARS-CoV-2	Citizens served by wastewater treatment plants in the cities of	Daily positive COVID-19 clinical tests and hospitalizations in	No sewage monitoring was conducted specifically for this study: "wastewater data was sourced from the KWR Water Institute" that conducted regular	KWR Water Research Institute	p

				Utrecht and Amsterdam	Utrecht and Amsterdam	sewage monitoring on a national level		
Swain et al. (53)	2023	United States of America	SARS-CoV-2	Students and staff at two universities, residents at psychiatric care facility, residents at senior living facility and students at a school	COVID-19 test results	Oakland County Health Division	Oakland County Health Division	e, f, s, t
Travis et al. (54)	2021	Unites States of America	SARS-CoV-2	Students at Hope College	Random surveillance testing data	Hope College	Hope College	e
Vo et al. (55)	2022	United States of America	SARS-CoV-2	Las Vegas metropolitan area	Clinical samples were collected and confirmed for the presence of SARS-CoV-2 RNA^	University of Nevada Las Vegas	Southern Nevada Health District	c
Wettstone et al. (64)	2023	Banglade sh	SARS-CoV-2	Citizens of Dhaka	Reported incidence of COVID-19 cases	University of Virginia in collaboration with Imperial College London and the International Centre for Diarrheal Disease Research Bangladesh	The general public, Directorate General of Health Services, Institute of Epidemiology, Disease Control and Research	e, f, g, n
White et al. (56)	2024	United States of America	SARS-CoV-2	Students at East Carolina University	COVID-19 test- and vaccination data	University of Carolina	University of Carolina	a, e, p

Wolken et al. (57)	2023	United States of America	SARS-CoV-2 and Influenza	Children attending preK-12 schools	Clinical COVID-19 testing data on students and staff, and community positivity rates for COVID-19, and citywide syndromic surveillance data on influenza	Not described	Houston Health Department	a, d, f, g
Wong et al. (65)	2021	Singapore	SARS-CoV-2	Residents living in an apartment building	Nasopharyngeal swab PCR^ tests, fever or respiratory symptoms	National Environment Agency Singapore and Ministry of Health Singapore	Ministry of Health Singapore	c, e, o
Wong et al. (66)	2024	Singapore	Zika virus	Singapore residents	Zika Virus test data and mosquito data	National Environment Agency of Singapore	Ministry of Health Singapore	m, p
Wurtz et al. (74)	2021	France	SARS-CoV-2	Citizens of Marseille	Confirmed COVID-19 cases	City of Marseille	Not described	p
Wurtzer et al. (75)	2020	France	SARS-CoV-2	Larger part of Paris	COVID-19 related hospitalizations, daily consultations and deaths	Not described	Not described	p
Yaari et al. (67)	2016	Israel	Poliovirus	Bedouin children living in the South of Israel	Stool survey data and vaccination data	Israeli government	Israeli Ministry of Health	f, v
Yaniv et al. (68)	2021	Israel	SARS-CoV-2	Citizens of Modi'in and Beer-Sheva	Morbidity data	Israeli Ministry of Health	Israeli Ministry of Health, and Ben-Gurion University of the Negev	p

*a: isolation or quarantine measures, b: hygienic measures, c: source tracing, d: contact tracing, e: test notification, f: vaccination campaign initiation, evaluation and/or (re)design g: public health messaging, promotion and/or education, h: (increased) community engagement, i: behavioral interventions, l: incorporation of sewage surveillance in regular surveillance, m: (improved) surveillance of (emerging) pathogen(s), n: enrichment of epidemiological data, o: implementation of sewage surveillance as early warning system, p: evaluation and/or adaptation of intervention(s), s: public health policy development, t: initiation or expanded collaboration with (public health) partners, u: resource allocation, v: expansion sewage surveillance, w: protect healthcare staff high at risk of severe disease

^SARS-CoV-2: severe acute respiratory syndrome coronavirus 2, RT-qPCR: real-time quantitative polymerase chain reaction, RNA: ribonucleic acid, PCR: polymerase chain reaction
